# Clinical significance of AGE-RAGE axis in colorectal cancer: associations with glyoxalase-I, adiponectin receptor expression and prognosis

**DOI:** 10.1186/s12885-016-2213-5

**Published:** 2016-03-01

**Authors:** Stratigoula Sakellariou, Paraskevi Fragkou, Georgia Levidou, Antonios N. Gargalionis, Christina Piperi, Georgia Dalagiorgou, Christos Adamopoulos, Angelica Saetta, George Agrogiannis, Irini Theohari, Stavros Sougioultzis, Panagiota Tsioli, Ioannis Karavokyros, Nikolaos Tsavaris, Ioannis D. Kostakis, Adamantia Zizi-Serbetzoglou, Gerasimos P. Vandoros, Efstratios Patsouris, Penelope Korkolopoulou

**Affiliations:** First Department of Pathology, “Laikon” General Hospital, University of Athens, Medical School, Mikras Asias 75, Goudi, 11527 Athens, Greece; Department of Biological Chemistry, University of Athens, Medical School, Athens, Greece; Department of Pathophysiology, “Laikon” General Hospital, University of Athens, Medical School, Athens, Greece; First Department of Surgery, “Laikon” General Hospital, University of Athens, Medical School, Athens, Greece; Department of Pathophysiology, Oncology Unit, “Laikon” General Hospital, University of Athens, Medical School, Athens, Greece; Second Department of Propedeutic Surgery, “Laiko” General Hospital, University of Athens, Medical School, Athens, Greece; Department of Pathology, “Tzaneio” General Hospital, Piraeus, Greece; Department of Pathology, “Agios Andreas” General Hospital, Patras, Greece

**Keywords:** AGE, RAGE, Glyoxalase-I, Adiponectin-receptor, Colorectal cancer

## Abstract

**Background:**

Advanced glycation end products (AGEs) and their receptor RAGE emerge as important pathogenic contributors in colorectal carcinogenesis. However, their relationship to the detoxification enzyme Glyoxalase (GLO)-I and Adiponectin receptors (AdipoR1, AdipoR2) in colorectal carcinoma (CRC) is currently understudied. In the present study, we investigated the expression levels of the above molecules in CRC compared to adjacent non-tumoral tissue and their potential correlation with clinicopathological characteristics and patients’ survival.

**Methods:**

We analyzed the immunohistochemical expression of AGE, RAGE, GLO-1, AdipoR1 and AdipoR2 in 133 primary CRC cases, focusing on GLO-I. The tumour MSI status was further assessed in mucinous carcinomas. Western immunoblotting was employed for validation of immunohistochemical data in normal and tumoral tissues as well in three CRC cell lines. An independent set of 55 patients was also used to validate the results of univariate survival analysis regarding GLO-I.

**Results:**

CRC tissue showed higher intensity of both AGE and RAGE expression compared with normal colonic mucosa which was negative for GLO-I in most cases (78 %). Western immunoblotting confirmed AGE, RAGE and GLO-I overexpression in tumoral tissue. GLO-I expression was directly related to RAGE and inversely related to AGE immunolabeling. There was a trend towards higher expression of all markers (except for RAGE) in the subgroup of mucinous carcinomas which, although of borderline significance, seemed to be more prominent for AdipoR1 and AGE. Additionally, AGE, AdipoR1 and Adipo R2 expression was related to tumor grade, whereas GLO-1 and AdipoR1 to T-category. In survival analysis, AdipoR2 and GLO-I overexpression predicted shortened survival in the entire cohort and in early stage cases, an effect which for GLO-I was reproduced in the validation cohort. Moreover, GLO-I emerged as an independent prognosticator of adverse significance in the patients’ cohort.

**Conclusions:**

We herein provide novel evidence regarding the possible interactions between the components of AGE-RAGE axis, GLO-I and adiponectin receptors in CRC. AGE and AdipoR1 are possibly involved in colorectal carcinogenesis, whereas AdipoR2 and GLO-I emerged as novel independent prognostic biomarkers of adverse significance for patients with early disease stage. Further studies are warranted to extend our observations and investigate their potential therapeutic significance.

## Background

Colorectal cancer (CRC) is the third most common malignancy in men and second in women, preceded by breast cancer in the latter [1]. The risk for CRC is significantly higher in obese and diabetic patients [2]. It is nowadays evident that environmental factors, such as a high caloric Western diet rich in fat, play an important role in the pathogenesis of the disease. Accumulating data suggest that insulin resistance, hyperglycemia and chronic inflammation lead to carcinogenesis through interaction with genetic characteristics, although the underlying molecular pathways remain to be clarified [2].

Advanced glycation end products (AGEs) are highly reactive molecules, formed endogenously or exogenously when glucose or other reducing sugars react with amino acids, nucleotide bases or fatty acids [3]. Endogenous AGEs are produced during metabolic oxidative stress and accumulate in tissues normally with age [4], their production dramatically accelerated by hyperglycemia [5]. High levels of exogenous AGEs, also known as glycotoxins, are generated by contemporary methods of cooking applying dry heat and high temperatures, beverages and cigarette smoking [3]. Exogenous AGEs are absorbed orally, thus directly influencing plasma concentrations and tissue deposition [6]. Excessive accumulation of glycotoxins has been implicated in many metabolic, degenerative and inflammatory disorders, as well as cancer development.

AGEs exert their detrimental effects by forming crosslinks with adjacent extracellular matrix proteins, thus reducing tissue flexibility [5]. Intracellular functions of AGEs are mediated via binding to the receptor for advanced glycation end products (RAGE), a transmembrane receptor and member of the immunoglobulin protein family. RAGE has been identified in a variety of cells and tissues [7] and is now considered a pattern recognition receptor (PRR) with a broad spectrum of ligands, including high-mobility group box-1 (HMGB1) and S100 proteins [8]. RAGE activation initiates downstream signaling pathways which promote inflammation, support cell survival, inhibit apoptosis and induce angiogenesis, leading to tumor growth and invasion in an inflammatory microenvironment [9]. Its expression has been detected in various tumors including colorectal carcinoma [10] and precancerous lesions such as colon adenomas [11]. Data derived from in vitro studies and rat models indicate that RAGE is significantly associated with CRC tumor development, progression and metastasis [12].

AGEs cytotoxicity is attenuated by the endogenous glyoxalase scavenging system [13]. Glyoxalase (GLO)-I and GLO-II are ubiquitously expressed enzymes which protect proteins, nucleotides and phospholipids from advanced glycation reactions by decreasing the levels of AGEs precursors [14]. Tumor cells with a high glycolytic rate benefit the most from the glyoxalase detoxification system. *GLO*-*I* gene amplification and overexpression has been identified in breast, lung, bladder, gastric and several other carcinomas [15]. With respect to CRC, amplification of *GLO I* is reported to be rare [15], although earlier studies demonstrated a 2-fold increased activity in tumor versus normal colonic epithelium [16].

Adiponectin, a peptide hormone produced exclusively by adipose tissue and implicated in glucose metabolism, appears to reduce insulin-resistance. Obese patients exhibit lower adiponectin serum levels compared to individuals of normal weight [17], whereas epidemiologic studies portray an inverse association between this hormone’s levels and various types of cancer, including CRC [18]. Adiponectin elicits its action through two receptors, AdipoR1 and AdipoR2, partly differing in distribution and potential functions [17]. Expression of AdipoR1 and AdipoR2 has been detected in pancreatic [19], gastric [20] and colon human carcinomas [21–25]. However, a few surveys dealing with CRC expression levels in relation to patients’ clinicopathological parameters, have resulted in inconclusive or controversial results.

Furthermore, adiponectin was found to be inversely associated with AGE/RAGE serum ratio [26] and similarly plasma levels of *N*-(carboxymethyl) lysine (CML), a prominent type of AGE, correlated negatively with adiponectin levels in overweight patients [27]. In vitro experiments support a link between AGE and adiponectin expression, implicating the anti-oxidative property of the latter [28] and arguing in favor of its protective effect on AGEs-induced endothelial dysfunction [27].

Current data on the role of the AGE-RAGE axis in CRC, particularly as related to GLO-I and AdipoR1or AdipoR2 expression, is insufficient. The present study was undertaken in an attempt to shed light upon the potential correlation between expression of the above molecules and clinicopathological characteristics, as well as the survival of CRC patients. To this end, we analyzed the expression of AdipoR1, AdipoR2, AGE, RAGE and GLO-I, focusing on the latter, in a large series of CRC patients with available clinicopathological characteristics and follow up and validated our findings by western immunoblotting in fresh tissue from normal colonic mucosa and CRC tissue, as well as in three colon cancer cell lines.

## Methods

### Patients

This is a retrospective study performed on archival tissue specimens from 133 cases of primary colorectal adenocarcinoma, diagnosed between 2000 and 2010 at the First Department of Pathology, University of Athens, Medical School, the Department of Pathology, “Tzaneio” General Hospital and the Department of Pathology, “Agios Andreas” General Hospital. The protocol of the study was approved by the University of Athens Bioethics Committee (PN 5106/2012) and follows the principles of the Declaration of Helsinki. Included were 61 men and 72 women with a median age of 70 years (range 41–87 years). Informed consent was obtained from all patients before their enrolment in the study. None of the patients had received chemotherapy or radiation before surgery.

All cases were reviewed by two experienced pathologists (PK, SS) for the assessment of the degree of differentiation, according to the latest World Health Organization classification of colorectal carcinoma. In order to assign a grade of differentiation in mucinous carcinomas (19 cases), the tumour MSI status was evaluated by both immunohistochemistry and molecular analyses. The nuclear expression levels of the respective MMR proteins were evaluated, whereas in those cases in which the immunohistochemical pattern could not define the MSI status, the molecular profile of additional MSI markers was identified (BAT25, BAT26, NR24, BRAF) [29]. Cases lacking mismatch repair (MMR) protein expression and therefore exhibiting MSI (MSI-high, MSI-H), were classified as low-grade carcinomas. On the other hand, tumours which did not exhibit MSI (MSI stable or MSI-low, MSS or MSI-L) were classified as high-grade carcinomas. Patient’s cohort consisted of 22 well differentiated (grade I), 90 moderately differentiated (grade II) and 21 poorly differentiated (grade III) adenocarcinomas.

According to the 6th edition of the TNM classification system, tumours were classified as stage I (*n* = 7 cases), stage II (*n* = 52 cases), stage III (*n* = 53 cases) and stage IV (*n* = 12 cases). In nine cases, corresponding to an incision tumour biopsy, assessment of TNM classification was not feasible. Patients’ clinicopathological characteristics are summarised in Table 1.

**Table 1:** Demographic characteristics of patients included in the study and validation cohort in this investigation

	Study cohort	Validation Cohort
Variable	Value	Value
Number of patients	133	55
Mean (range):		
Patients age, years	70 (41–87)	74.5 (42–90)
Follow-up, months	44 (1–84)	26 (1–60)
	n (%)	n (%)
Gender:		
Female	72 (54.13 %)	28 (50.91 %)
Male	61 (45.87 %)	27 (49.09 %)
Histological Grade		
I	22 (16.54 %)	3 (5.45 %)
II	90 (67.67 %)	36 (65.45 %)
III	21 (15.79 %)	16 (29.09 %)
Tumor status		
T1	11 (8.27 %)	2 (3.64 %)
T2	12 (9.02 %)	12 (21.82 %)
T3	87 (65.42 %)	33 (60.00 %)
T4	23 (17.29 %)	8 (14.55 %)
Nodal status		
No	62 (46.62 %)	32 (58.18 %)
N1	36 (27.07 %)	15 (27.27 %)
N2	25 (18.79 %)	8(14.55 %)
Not available	10 (7.52 %)	-
TNM stage		
I	7 (5.26 %)	10 (18.18 %)
II	52 (39.09 %)	19 (34.55 %)
III	53 (39.86 %)	17 (30.91 %)
IV	12 (9.02 %)	9 (16.36 %)
Not available	9 (6.77 %)	-
Tumor location		
Right colon	54 (40.60 %)	21 (31.18 %)
Left colon	79 (59.40 %)	34 (61.82 %)
Histological type		
Mucinous	19 (14.29 %)	5 (9.09 %)
Non-mucinous	114 (85.71 %)	50 (90.91 %)

By the time this study was undertaken, 35 patients had died of their disease after a median survival of 19 months (range 1–57) and nine were lost to follow-up. The median follow-up for the remaining 89 patients was 56 months (range 12–84 months). Information regarding recurrence was available in 119 patients, among which 27 had recurred.

### Immunohistochemical analysis

Immunostaining for AGE, RAGE, Glyoxalase-I, Adiponectin Receptor Type 1 and Adiponectin Receptor Type 2 was performed on freshly cut, paraffin-embedded 4 μm sections of formalin fixed tumour tissue which were left to dry overnight at 37 °C using the two-step peroxidase conjugated polymer technique (DAKO Envision kit, DAKO, Carpinteria, CA). For antigen retrieval, tissue sections were treated in the microwave with sodium citrate buffer, pH 6.0, for 20 min. Sections were incubated with the primary antibodies overnight at 4 °C, except for the anti-Adiponectin Receptor Type 1 antibody, where the incubation time was 60 min at 4 °C. Details on the primary antibodies used are listed in Table 2. In negative controls the primary antibody was substituted by nonimmune serum.

**Table 2:** Characteristics of antibodies used

Antibody	Supplier/Company	Methods of antigen retrieval	AB dilution	Conditions of incubation
Anti-AGE	TransGenic	Microwave 20 min	1:500	Overnight
(monoclonal mouse)		Citrate buffer (pH6)		4 °C
Anti-RAGE	R&D	Microwave 20 min	1:500	Overnight
(polyclonal goat)		Citrate buffer (pH6)		4 °C
Anti-Glyoxalase-I	Santa Cruz	Microwave 20 min	1:300	Overnight
(monoclonal mouse)	Biotechnology	Citrate buffer (pH6)		4 °C
Anti-Adiponectin Receptor Type 1	Phoenix	Microwave 20 min	1:500	60 min
(polyclonal rabbit)	Pharmaceuticals	Citrate buffer (pH6)		4 °C
Anti-Adiponectin Receptor Type 2	Phoenix	Microwave 20 min	1:500	Overnight
(polyclonal rabbit)	Pharmaceuticals	Citrate buffer (pH6)		4 °C

Immunohistochemical evaluation was performed by two pathologists (PK, PF), without knowledge of the clinical information. For each antibody, the staining distribution (cytoplasmic, nuclear, membranous, luminal) as well as the staining pattern (diffuse or granular) was recorded. The percentage of positive neoplastic cells as well as the intensity of staining (1: faint staining, 2: moderate staining, 3: intense staining) were evaluated separately. Moderate staining was defined as equivalent to that of adjacent lymphocytic aggregates. A Histo-score (H-score) based on the percentage of stained neoplastic cells multiplied by staining intensity was calculated. Finally, in order to investigate any differences in the expression of AGE, RAGE, AdipoR1 and AdipoR2 between the normal and neoplastic tissue, the extent and the intensity of staining observed in the normal adjacent colonic mucosa (in those cases in which it was available) was also recorded.

### Western immunoblotting

Protein extraction from two normal colon mucosa, 2 CRC neoplastic tissues and three colon carcinoma cell lines (SW480, HT29, HCT116) was performed using ice-cold RIPA lysis buffer (#9801, Cell Signaling Technology) containing protease and phosphatase inhibitors with the extra addition of PMSF (Sigma Aldrich). Bradford assay (Bio-Rad) was used to assess protein concentration in the extracts. Proteins were separated by SDS-polyacrylamide gel electrophoresis and transferred to a nitrocellulose membrane (Porablot NCP, Macherey-Nagel). Membranes were blocked for 1 h in room temperature in Phosphate Buffered Saline Tween-20 (PBST) with 5 % non-fat milk. Subsequently, membranes were incubated overnight at 4 °C with the following primary antibodies: anti-AGE (Abcam, ab23722, dilution 1:1000), anti-RAGE (Santa Cruz Biotechnology, sc-8230, dilution 1:200), anti-glyoxalase (GLO-I) (sc-133144, dilution 1:200). Primary antibodies were diluted in PBST containing 1 % non-fat milk. The membranes were then incubated with the HRP - conjugated secondary antibodies for 1 h in room temperature. Secondary antibodies goat anti-rabbit IgG-HRP (12–348, Millipore) and goat anti-mouse IgG-HRP (12–349, Millipore) diluted 1:2000 were used. The detection of the immunoreactive bands was performed with the SuperSignal WestPico Chemiluminescent HRP Substrate kit (Thermo Scientific). Relative protein amounts were evaluated by a densitometric analysis using Image J software (La Jolla, CA, USA) and normalized to the corresponding Actin levels. All experiments have been performed at least 3 times and representative results of one experiment are shown.

### Statistical analysis

AGE, RAGE, AdipoR1 and AdipoR2 were treated as continuous variables. Associations with tumor stage, grade and histological type were evaluated using non-parametric tests, with correction for multiple comparisons (Bonferroni correction) when necessary (Mann Whitney *U* test, Kruskal Wallis ANOVA, Spearman’s correlation coefficient and Fischer’s exact test).

Survival analysis was performed using death due to CRC as the end-point. The effect of various parameters (age, sex, tumor location, stage, grade and AGE, RAGE, AdipoR1 and AdipoR2 expression) on clinical outcome was assessed by plotting survival curves according to the Kaplan–Meier method and comparing groups using the log rank test, as well as with multivariate analysis (Cox's model). In survival analysis (univariate and multivariate), continuous variables were categorized on the basis of results obtained from ROC (Receiver Operating Characteristic) curves. Univariate survival analysis stratified by TNM status was also performed for the examined parameters, in order to adjust their effect on survival for tumour stage. Multivariate survival models were adjusted for those parameters that were proven to be significant in univariate analysis, in order to evaluate the predictive power of each parameter independently of the others. Statistical calculations were performed using the Statistical package STATA 11.0 for Windows (Stata Corp., College Station, TX, USA). All results with a two-sided p level ≤ 0.05 were considered of marginal significance.

### Validation cohort

An independent set of patients with CRC was used to validate the chosen cut-off values for the expression of GLO-I in univariate analysis. The results of univariate survival analysis for GLO-1 expression in the population group were used to calculate the required number of patients in the validation group for an adequately powered analysis (80 %). In order to detect a Hazard ratio of 2.8836 as calculated in the patients’ group- using a two-sided log-rank test and to achieve 80 % power at a 0.05 significance level, 47 patients would be needed.

The validation group we used consisted of 55 patients, the demographic data of whom are shown in Table 1. Patients were diagnosed at the First Department of Pathology, University of Athens, Medical School, the Department of Pathology, “Tzaneio” General Hospital and the Department of Pathology, “Agios Andreas” General Hospital and treated at “Laikon” General Hospital, University of Athens, “Tzaneio” General Hospital and “Agios Andreas” General Hospital, between 2010 and 2014.

## Results

### AGE and RAGE expression in normal colonic tissue, colorectal adenocarcinomas and colon cell lines

**Fig. 1:**
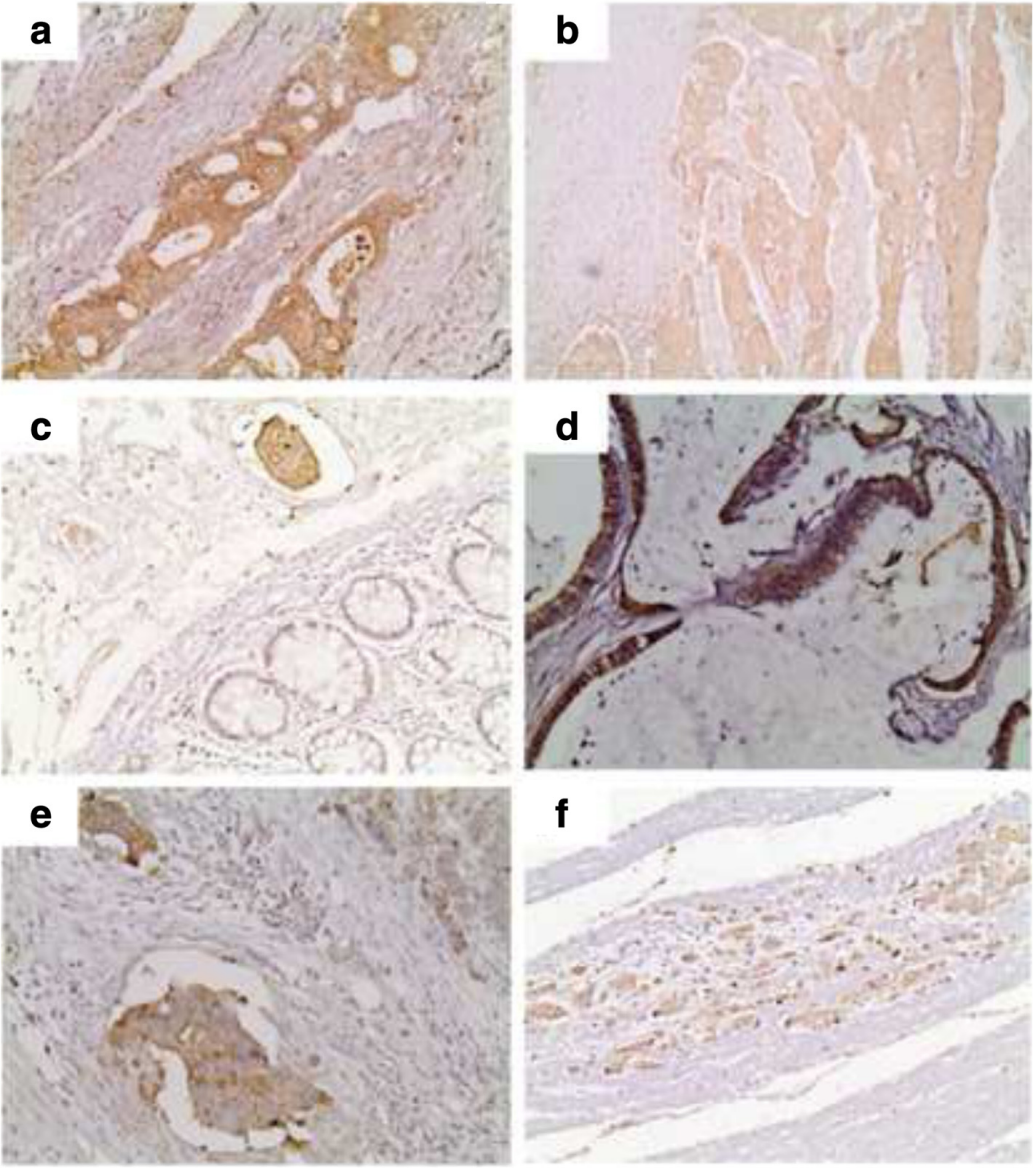
AGE (aX20, bX10) and RAGE (c–f X20) immunoreactivity in CRC. Lower AGE expression is seen in a grade III (**b**) versus a grade II (**a**) adenocarcinoma. RAGE expression is higher in tumoral tissue versus normal colonic mucosa (**c**) as well as in mucinous adenocarcinoma (**d**) whereas low expression is depicted in a grade II adenocarcinoma (**f**)

AGE expression was observed in 122/124 examined cases (98.39 %) with H-score ranging between 0 and 300 (median: 90). Accordingly, RAGE was expressed in all but one cases (124/125, 99 %), with H-score ranging between 0 and 285 (median: 100). Both molecules had a diffuse cytoplasmic staining pattern (Fig. 1a–f).

**Fig. 2:**
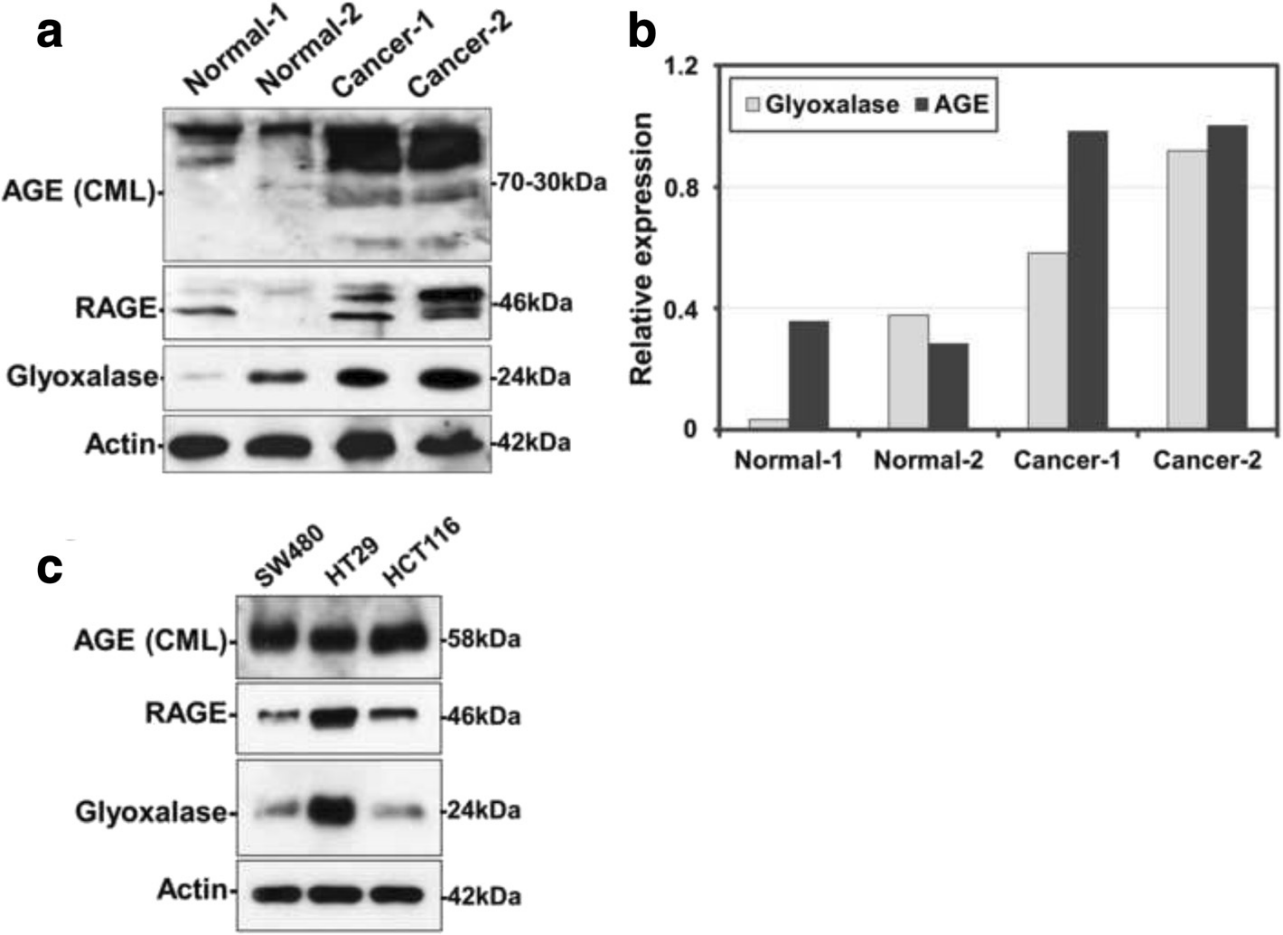
Western immunoblot indicating AGEs, RAGE and GLO-I protein expression in two CRC and adjacent normal tissues **a**. The densitometric quantification of AGEs and GLO-I expression (normalized to the actin levels) is shown in the graph **b**. Western immunoblot indicating AGEs, RAGE and GLO-I protein expression in tree colon cancer cell lines SW480, HT29 and HCT116 **c** All experiments have been performed at least three times and representative results of one experiment are shown

AGE and RAGE immunoreactivity in the adjacent normal colonic tissue was feasible in 89 cases for both antibodies. Although there was no difference in the extent of AGE and RAGE immunoexpression between normal and neoplastic areas, the intensity of both AGE and RAGE expression was higher in the tumoral tissue (*p* = 0.0001 for both relationships) Fig. 1c. Validation of the immunohistochemical data by western immunoblotting revealed that AGE and RAGE expression was higher in CRC tissue compared to adjacent normal colon in the two tested cases (Fig. 2).

Furthermore, western blot analysis of colon cancer cell lines, SW480 colon adenocarcinoma, HT29 colon adenocarcinoma grade II, HCT116 colon carcinoma revealed high protein expression of AGEs and RAGE (Fig. 2c).

**Fig. 3:**
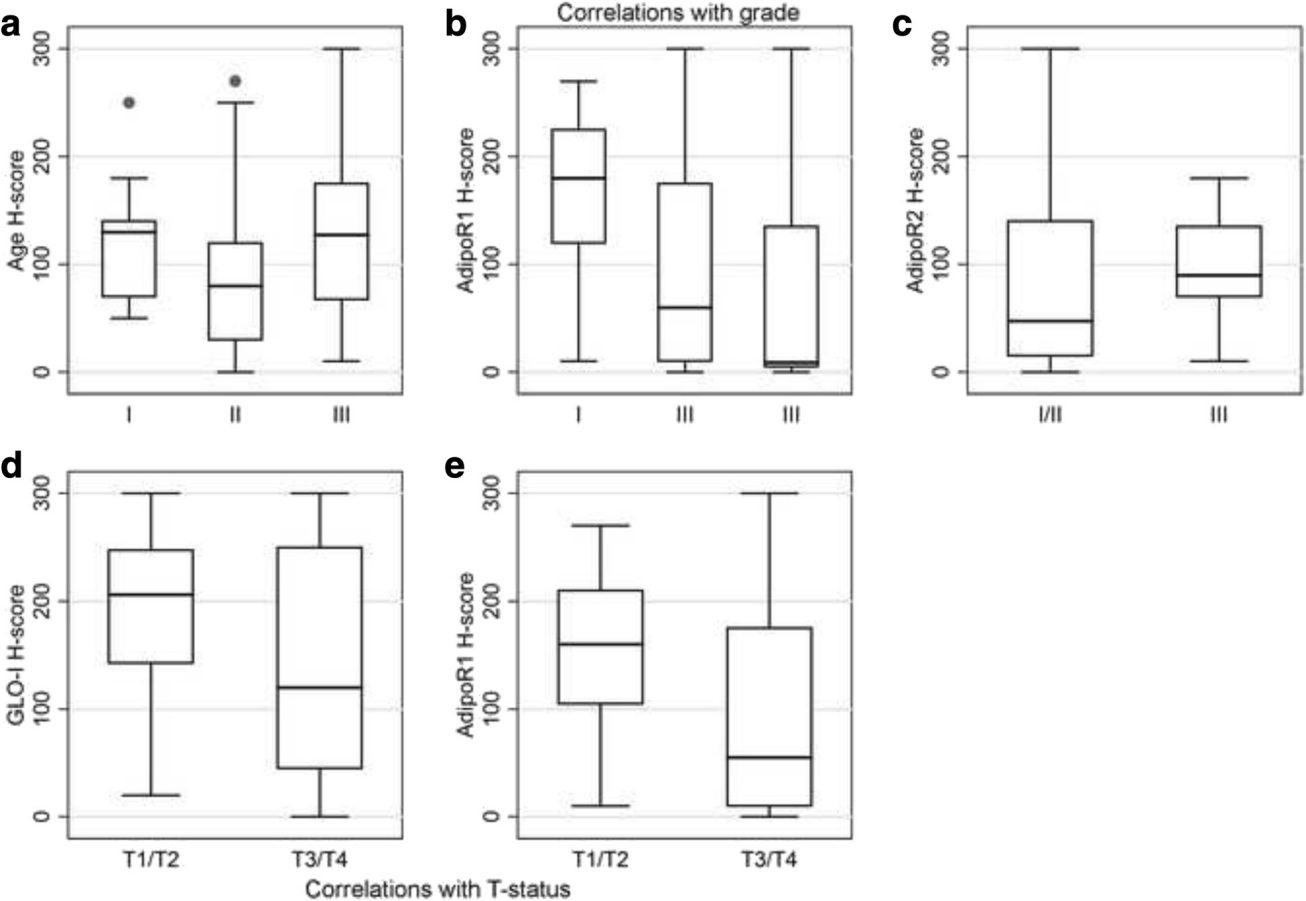
**a** Box plot illustrating the association of AGE expression with grade. **b** Box plot illustrating the inverse association of AdipoR1 H-score with grade. **c** Box plot illustrating the inverse association of AdipoR2 H-score with grade. **d** Box plot illustrating the inverse association between GLO-I immunoexpression and T-status. **e** Box plot illustrating the inverse association of AdipoR1 H-score with T-status

Intriguingly, mucinous adenocarcinomas presented marginally higher AGE expression (Mann–Whitney *U* test, *p* = 0.0997, Fig. 1d). Moreover, AGE H-score was correlated with tumor histological grade (Mann–Whitney *U* test I vs II vs III, *p* = 0.0157, Fig. 1a, b and Fig. 3a, Table 3), the lower levels being observed in grade II cases. No correlations were observed between AGE or RAGE and tumor stage or location (*p* > 0.10).

**Table 3:** Distribution of AdipoR1, AdipoR2, AGE, RAGE, GLO-I expression (%) and H-score among different groups of histological grade, TNM status, T - category, Nodal metastasis status and histological type

		AdipoR1	AdipoR2	AGE	RAGE	GLO-I
		median (range)	median (range)	median (range)	median (range)	median (range)
Histological grade						
I	%	80 (10–90)	30 (10–95)	70 (20–100)	80 (30–100)	90 (5–100)
	H-score	120 (10–270)	60 (10–237.5)	110 (35–250)	120 (30–255)	225 (10–300)
II	%	40 (0–100)	40 (0–100)	50 (0–100)	65 (0–100)	70 (0–100)
	H-score	60 (0–300)	45 (0–300)	80 (0–270)	100 (0–247.5)	136.25 (0–300)
III		7.5 (0–100)	80 (20–90)	70 (0–100)	80 (10–100)	100 (0–100)
		8.75 (0–300)	90 (10–180)	120 (0–300)	100 (10–285)	200 (0–300)
TNM status						
I/II	%	55 (0–100)	40 (0–100)	60 (0–100)	75 (5–100)	80 (0–100)
	H-score	60 (0–300)	57.5 (0–300)	90 (0–300)	112.5 (5–285)	140 (0–300)
III/IV	%	60 (0–100)	65 (0–100)	60 (0–100)	60 (0–100)	70 (0–100)
	H-score	105 (0–300)	60 (250)	83.75 (0–270)	90 (0–250)	132.5(0–300)
T-category						
T1/T2	%	70 (10–90)	40 (20–95)	90 (10–250)	80 (0–100)	90 (20–100)
	H-score	160 (10–270)	60 (30–237.5)	70 (10–100)	90 (0–200)	206.25(20–300)
T3/T4	%	45 (0–100)	50 (0–100)	90 (0–300)	70 (10–100)	70 (0–100)
	H-score	55 (0–300)	60 (0–300)	60 (0–100)	105 (10–285)	120(0–300)
Nodal metastasis						
Absent	%	55 (0–100)	45 (0–100)	60(0–100)	80(5–100)	80(0–100)
	H-score	75 (0–300)	70 (0–300)	90(0–300)	120(5–285)	140(0–300)
Present	%	60 (0–100)	55 (0–100)	60(0–100)	60(0–100)	70 (0–100)
	H-score	105 (0–300)	60 (0–250)	80(0–270)	90(0–250)	122.5(0–300)
Histological type						
Mucinous	%	80 (5–100)	70 (15–90)	60(0–100)	85(3–100)	95(10–100)
	H-score	150 (5–300)	70 (12–180)	120(0–300)	120(60–285)	125 (15–300)
Non-mucinous	%	50 (0–100)	40 (0–100)	60(0–100)	60(5–100)	75(0–100)
	H-score	60 (0–300)	47.5 (0–300)	80(5–270)	100(5–250)	140(0–300)

### GLO-I expression in normal colonic tissue, colorectal adenocarcinomas and colon cancer cell lines

**Fig. 4:**
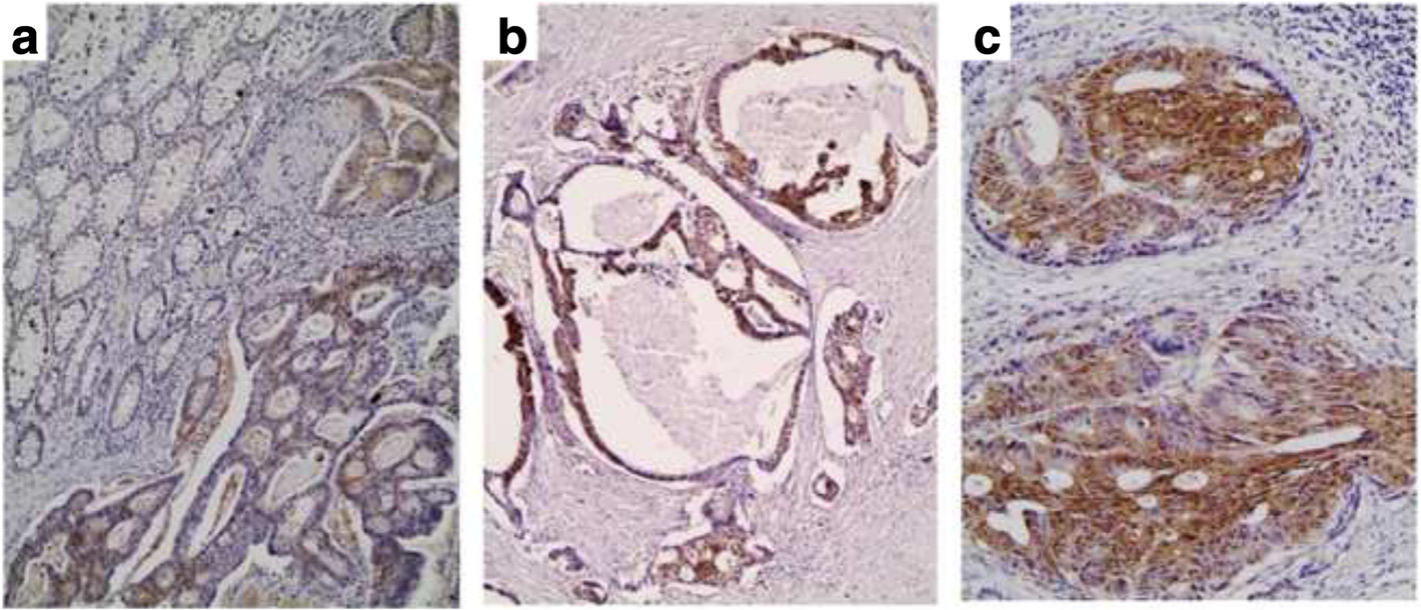
GLO-I immunoreactivity in CRC (aX10, bX10, cX20). Normal colonic mucosa is negative for GLO-I (**a**), whereas mucinous adenocarcinomas display high GLO-I H-scores (**b**)

Cytoplasmic GLO-I expression (Fig. 4a–c) was observed in 94.17 % of the examined cases (113/120). The median H-score was 140 (range 0–300).

Normal adjacent colonic mucosa was mostly negative for GLO-I, with 20 of the examined cases (20/91, 22 %) being faintly to moderately positive (Fig. 4a). Furthermore, although it was unrelated to tumor histological grade (*p* > 0.10), GLO-I expression was negatively correlated with tumor T-category (Mann–Whitney *U* test T1/T2 vs T3/T4, *p* = 0.0246, Fig. 3d, Table 3). Investigation of GLO-I expression by western immunoblotting in normal and CRC samples, revealed higher protein expression in tumor tissue (Figs. 2 and 4a). Similarly, colon cancer cell lines revealed high GLO-I expression (Fig. 2c). An inverse relation between GLO-I and AGE expression was clearly observed in normal colonic mucosa and colon cancer cell lines, while it was less evident in CRC tissue at both immunohistochemical and western blot analysis.

Significant positive correlations emerged between GLO-I and RAGE or Adipo1 H-score (Spearman’s correlation coefficient, *R* = 0.2909, *p* = 0.0017 for the former relationship and *R* = 0.4096, *p* = 0.0001 for the latter). On the other hand, an inverse relationship emerged between GLO-I and AGE, with increased levels of GLO-I H-score (≥140) being correlated with lower AGE H-score (Mann–Whitney *U* test, *p* = 0.0731), a relationship of borderline significance however.

### AdipoR1 and AdipoR2 expression in normal colonic tissue and colorectal adenocarcinomas

**Fig. 5:**
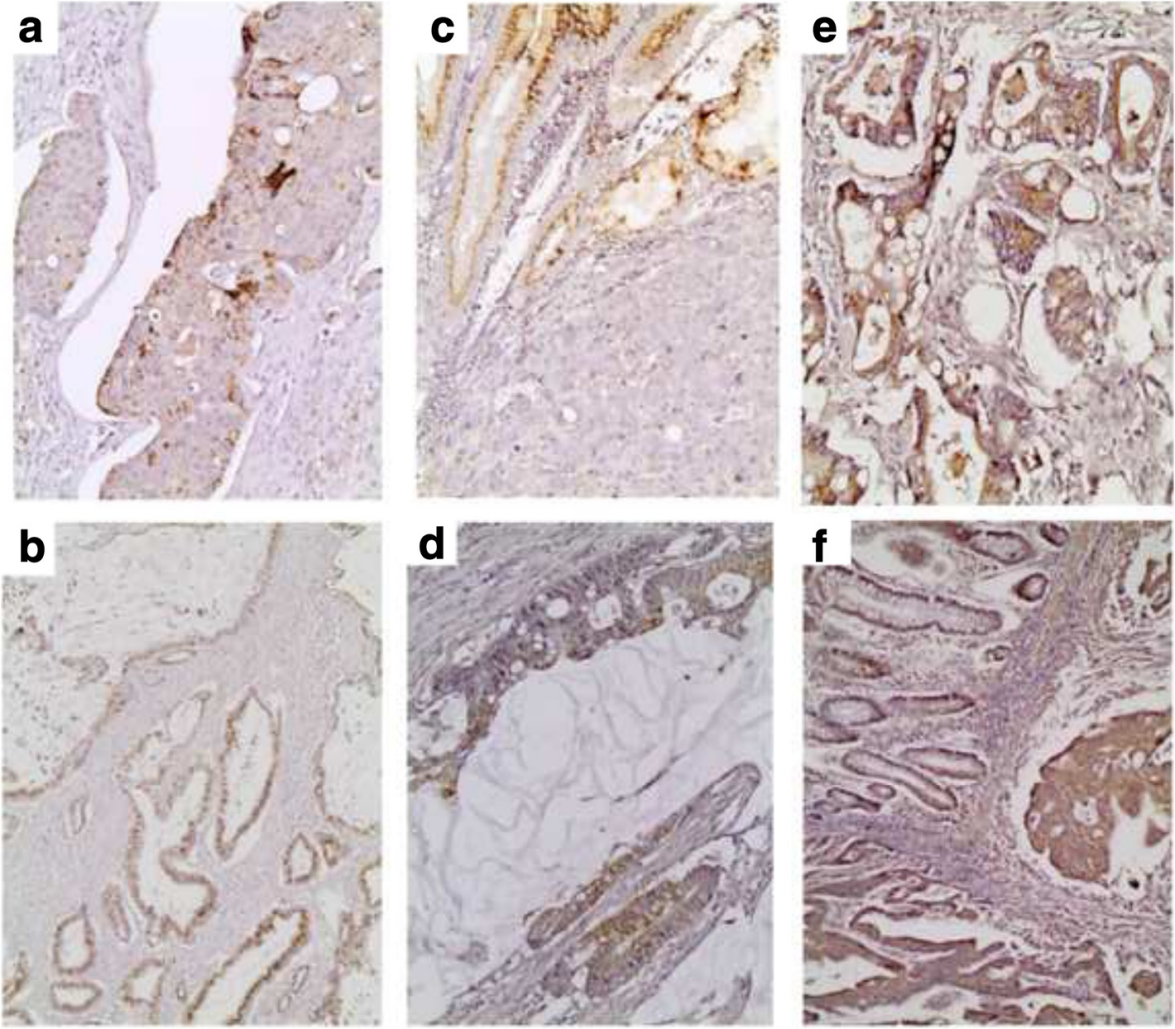
AdipoR1 (**a**–**d**) and AdipoR2 (**e**, **f**) immunohistochemistry in CRC. Normal colonic mucosa is strongly positive for AdipoR1as opposed to adjacent CRC (Note the cytoplasmic granular staining more prominent near the luminal surface) (**c**). Mucinous adenocarcinomas display high levels of AdipoR1 (**b**) and AdipoR2 (**e**). Lower grade tumors display higher levels of AdipoR1 (d *vs* c). No difference is observed between normal and CRC tissue for AdipoR2 expression (**f**). (aX20, bX20, cX20, dx20, eX20, fX10)

AdipoR1 and AdipoR2 immunoreactivity was observed in the majority of the examined cases (93/100, 93 % and 90/96, 93.7 % respectively). Both proteins had a wide range of H-score (0–300, median value: 85 and 60 respectively) and a cytoplasmic (Fig. 5a–f), often granular staining, more prominent near the luminal surface (Fig. 5c).

The adjacent normal colonic tissue displayed marginally more intense AdipoR1 expression when compared to the neoplastic tissue in the 70 cases in which it could be evaluated (Mann Whitney *U* test, *p* = 0.0530) (Fig. 5c). On the contrary, there was no difference in AdipoR2 expression between the normal and neoplastic areas (the former evaluated in 70 cases) (*p* > 0.10) (Fig. 5).

Mucinous adenocarcinomas had marginally higher AdipoR1 and AdipoR2 expression levels (Mann–Whitney *U* test, *p* = 0.0739 and 0.0993) (Fig. 5b, e). Moreover, AdipoR1 H-score was negatively correlated with tumor histological grade (Fig. 5a–d), (Kruskal Wallis ANOVA, I vs II vs III, *p* = 0.0080, Fig. 3b, Table 3) and T-category (Mann Whitney *U* test, T1/2 vs T3/4, *p* = 0.0363, Fig. 3e). On the contrary, grade III tumors tended to have higher levels of AdipoR2 expression when compared to lower grades (I/II) [Mann Whitney *U* test, *p* = 0.0210 (Fig. 3c). The correlations between AdipoR1 or AdipoR2 and tumor TNM stage or location were not significant (*p* > 0.10).

### Survival analysis

**Fig. 6:**
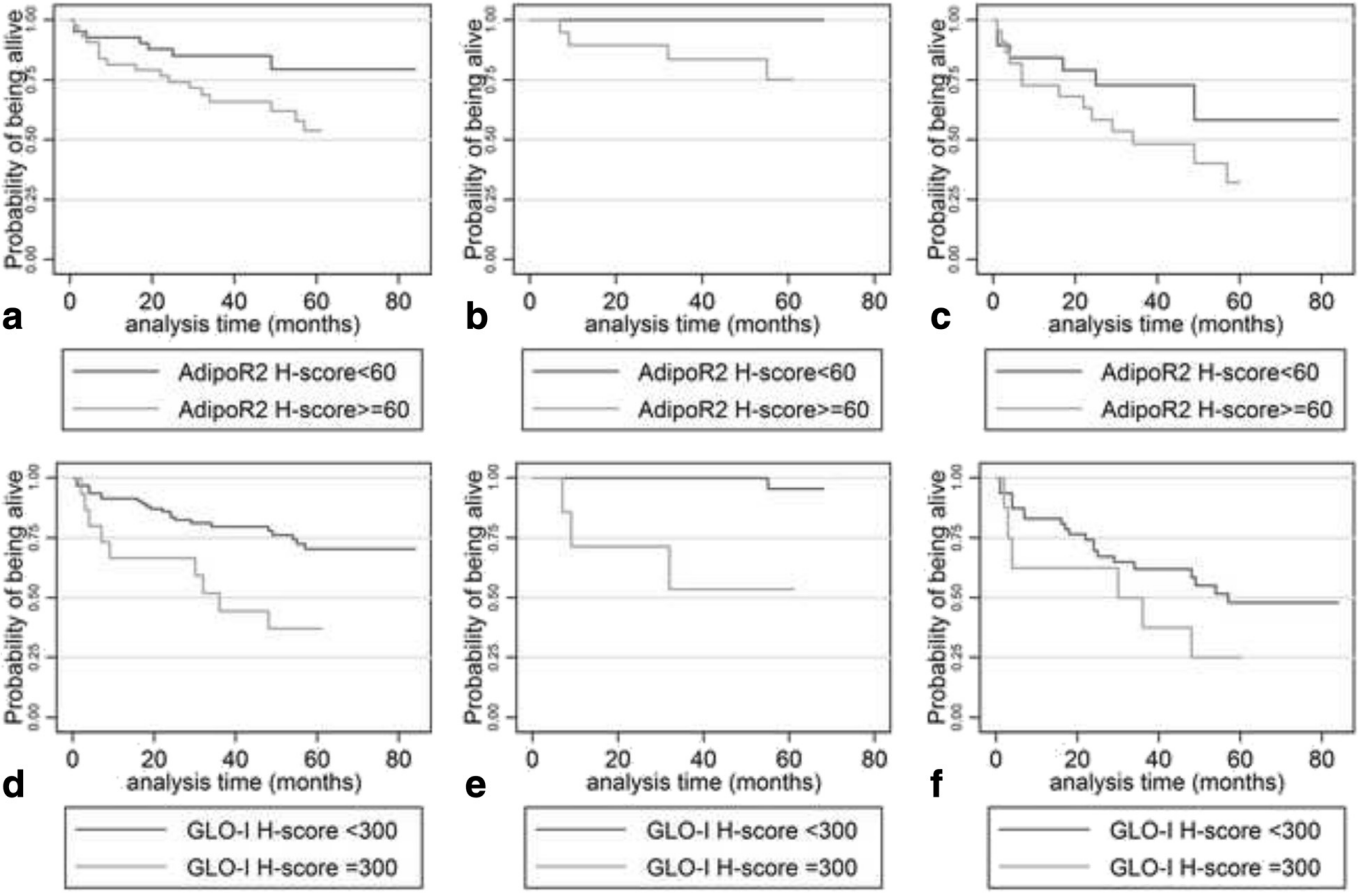
Kaplan-Meier curves illustrating the adverse prognostic significance of AdipoR2 (**a**, **b**, **c**) and GLO-I (**d**, **e**, **f**) H-score in the whole population cohort (**a**, **d**) as well as in early (**b**, **e**) and advanced stage (**c**, **f**) cases

Univariate survival analysis showed that advanced tumor stage according to TNM status (I/II vs III/IV, *p* = 0.0001), the presence of nodal (N) metastasis (*p* = 0.0005), the presence of distant metastasis (M) (*p* = <0.0001), increased AdipoR2 H-score (<60 vs ≥60) *p* = 0.0465, Fig. 6a), as well as increased GLO-I H-score (300 vs <300, *p* = 0.0046, Fig. 6d), were all correlated with shortened survival (Table 4). Stratified survival analysis according to TNM status showed that the effect of both AdipoR2 and GLO-I on patients’ survival was significant in early stage tumours (TNM stage I/II, *p* = 0.0002 for AdipoR2, Fig. 6b, and *p* = 0.0499 for GLO-I, Fig. 6e) in contrast to advanced stage ones (TNM stage III/IV), in which both proteins failed to retain their statistical significance (Fig. 6c, f). AdipoR1, AGE and RAGE did not add significant prognostic information in this regard.

**Table 4:** Results of univariate survival analysis regarding overall survival and recurrence-free survival in patients with colorectal carcinoma

	Overall survival p-value
Age	
< 70 vs ≥70 years	>0.10
Gender	
Male vs female	>0.10
Histological type	
non mucinous vs mucinous	>0.10
Histological grade	
I vs II vs III	0.0721
TNM stage	
I vs II vs III vs IV	0.0001
Tumour status	
T1-T2 vs T3-T4	>0.10
Nodal status	
No vs N1 vs N2	0.0005
AdipoR1 H-score	
< 85 vs ≥85	>0.10
AdipoR2 H-score	
< 60 vs ≥60	0.0465
AGE H-score	
< 90 vs ≥90	>0.10
RAGE H-score	
< 100 vs ≥100	>0.10
GLO-I H-score	
< 300 vs ≥300	0.0046

In multivariate survival analysis, after adjusting the effect of GLO-I or AdipoR2 for TNM status in two separate Cox regression models, this remained significant, supporting a TNM-independent prognostic role for both molecules (HR = 2.76, *p* = 0.010 for GLO-I and HR = 2.53, *p* = 0.050 for AdipoR2). Interestingly, in a multivariate model including all three parameters that were proven to be significant in univariate analysis (GLO-I, AdipoR2 and TNM status), GLO-I emerged as an independent predictor of adverse prognosis (HR = 2.98, *p* = 0.036), along with TNM status (HR = 7.53, *p* < 0.0001).

#### Survival analysis-validation group

**Fig. 7:**
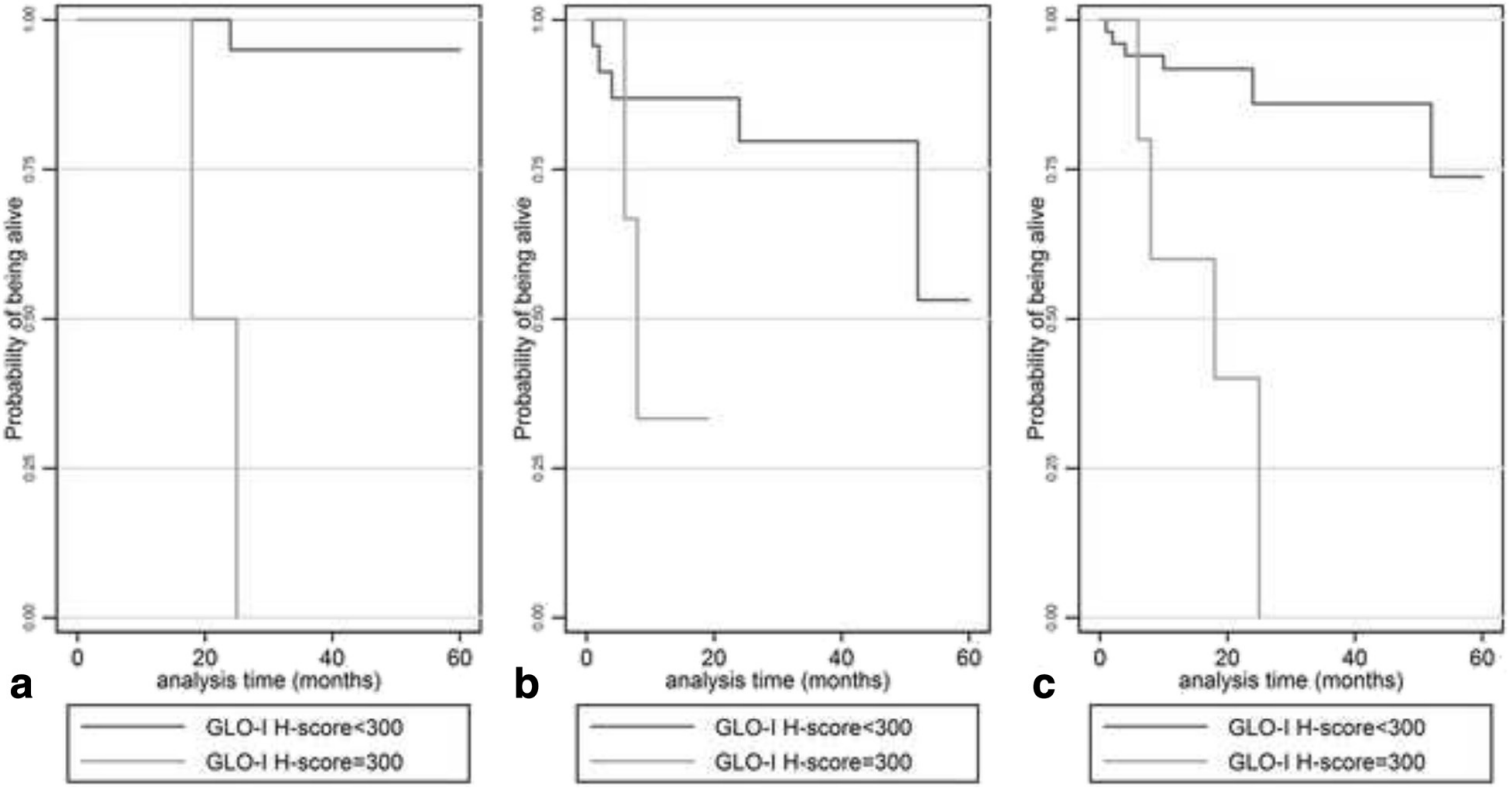
Kaplan-Meier curves illustrating the adverse prognostic significance of GLO-I H-score in the entire validation cohort (**a**) as well as in early (**b**) and advanced stage (**c**) cases

Univariate survival analysis in this group of patients showed that overall survival was significantly lower in the GLO-I low-expressor compared to the GLO-I high-expressor group (H-score 300 vs <300, log-rank test, *p* < 0.0001, Fig. 7a). This effect remained significant in early stage cases (TNM stage I/II, log-rank test *p* < 0.0001, Fig. 7b), while it was of borderline significance in advanced stage cases (TNM stage III/IV, log-rank test *p* = 0.0527, Fig. 7c).

## Discussion

The present cohort examined the immunohistochemical localization of AGEs and their receptor RAGE expression in CRC versus adjacent non-tumoral tissue. Furthermore, the expression of the detoxification enzyme GLO-I and AdipoR1, AdipoR2 was also evaluated in an attempt to reveal probable interrelations, associations with clinicopathological characteristics or potential impact on survival of CRC patients.

Previous studies in cancer cell lines and animal models point towards the implication of the AGE-RAGE axis in CRC development. Our western blot analysis in 3 colon cancer cell lines reaffirms this notion, documenting an increased expression of AGE and RAGE. Kuniyasu et al. [12] first showed that RAGE was expressed in all examined CRC cell lines and its expression was associated with in vitro cancer cell migration and invasion. In the same study, cell treatment with AGE-bovine serum albumin partially affected cell growth, migration and invasion, implying a minimal direct role of AGEs in CRC progression. In another survey, RAGE expression was documented in Caco-2 human colon carcinoma cell line, and treatment with AGE resulted in p44/42 (ERK1/2) MAP kinase activation in a time- and dose-dependent manner [30]. Furthermore, Turovskaya et al. [31] showed that RAGE promoted molecular tumor-stromal interactions in a colitis-associated carcinogenesis mouse model leading to colon tumor development. Similarly, it has been hypothesized that RAGE acting synergistically with the epithelial to mesenchymal transition pathway can trigger the production of cancer stem cells [32].

Despite vigorous experimental research in the field, data remain scarce regarding AGE and RAGE expression in human normal versus tumorous colorectal epithelium. In the present study, both AGE and RAGE tended to be more intense in tumoral compared to non-tumoral epithelium, also validated by western immunoblotting in two normal and two cancerous tissues. In this context, Zill et al. [30] observed RAGE expression in non-neoplastic human intestinal tissues by RT-PCR. Kuniyasu et al. [33] reported absence of RAGE mRNA in normal colon mucosa in three examined cases. In the same study, immunohistochemical analysis of a large number of CRC samples failed to reveal AGE positivity in the normal colonic mucosa. The above inconsistencies may be attributed to variability of applied methodology and/or different antibodies usage. However, further research is warranted to clarify this issue. Notably, in an experimental survey by Heijmans et al. [34], RAGE positivity was demonstrated throughout the normal intestinal epithelium of the mice under study.

In our cohort, we found that AGE expression was associated to cancer histological grade, with lower levels being observed in grade II cases. This finding suggests a possible role during the early and late stages of colorectal carcinogenesis. AGE expression has not been previously reported to correlate with CRC grade. However, in gastric cancer patients, Kuniyasu et al. [35] found RAGE positivity to be directly associated with tumor grade. In our survey, AGE and RAGE were not related to stage or any of its parameters, despite previous studies presenting a significant positive correlation between RAGE and lymph node as well as distant metastasis [33].

Regarding the endogenous detoxification enzyme of AGEs, our immunohistochemical and western blot analysis are in agreement with previous reports showing that GLO-I is more frequently expressed in CRC than adjacent normal epithelium [16]. In addition, our results confirmed the expected inverse relation between GLO-I and AGE expression by western blotting in normal colonic mucosa and colon cancer cell lines. However, this relationship was not as clear in CRC tissue at both the immunohistochemical and western blot level. These findings overall recapitulate the attenuation of AGEs cytotoxicity by the Glyoxalase scavenging system [13]. High GLO-I immunoreactivity cases also showed high RAGE positivity, possibly reflecting an adaptation mechanism, with cancer cells exhibiting RAGE up-regulation to overcome the GLO-I induced decrease of AGE levels. The above findings attribute a significant role to the AGE-RAGE-GLO-I system in CRC. In the present cohort, tumors with high GLO-I expression were more frequently of low T category.

Furthermore, AdipoR1 and R2 were expressed both in CRC and normal colonic mucosa. AdipoR1 immunostaining tended to be more intense in the latter, while no significant difference was observed regarding the percentage of positive cells in neoplastic *vs* non-neoplastic epithelium. In accordance with our findings, recent surveys report AdipoR1 and R2 detection in cancerous as well as normal colonic tissue, although there is disagreement concerning the expression levels. Gialamas et al. [21] and Williams et al. [24] using IHC, and Yamamoto et al. [25] by performing RT-PCR, found a higher expression of AdipoR1 and R2 in CRC than in normal colonic epithelium [21, 24, 25]. On the contrary, in a recent survey from Japan [23], AdipoR1 and R2 mRNA levels were significantly lower in neoplastic tissue compared to normal. Based on the above results, the authors suggested that reduction of AdipoR permits cancer cells to escape serum adiponectin tumor suppressive effects. Since it is difficult to explain the above differences, larger prospective studies are required to comparatively evaluate AdipoR1, R2 protein and mRNA expression in normal, dysplastic and cancerous colon tissues.

In our cohort, AdipoR1 immunopositivity was inversely related to tumor histological grade and T category, in agreement with previous reports [22]. Concurrent with these findings, a prognostic role of serum adiponectin in CRC patients is suggested, since this hormone’s concentration has been found to be negatively correlated with CRC stage [18, 36], and a high leptin/adiponectin ratio has been reported as a predictor for adverse outcome [37]. Adiponectin serum levels were also inversely related to CRC grade in one study [21]. On the contrary, in another report [38], the hormone’s immunohistochemical expression by colon neoplastic tissue showed a positive association with tumor grade. Recent investigation in various cancer cell lines has shown that adiponectin possesses both anti-proliferating and pro-apoptotic effects [22, 39, 40]. Concerning CRC cell lines, growth inhibition and apoptosis induction after adiponectin treatment has been attributed to MAPK/mTOR pathway activation [41, 42]. Moreover, recent investigations have shown reduced AdipoR1 expression in tumors with lymph node metastasis [21, 23] and venous invasion [25]. The above findings support AdipoR1 overexpression as an early event in CRC tumorigenesis, also suggesting a protective role against tumor progression.

Regarding AdipoR2, we found a positive association with grade. Grade III tumors showed higher levels of expression than grade I/II, a finding in discordance to Byeon et al. [22]. However, no significant relation to stage or any staging parameters was observed. Interestingly, literature regarding AdipoR2 and stage appears to be controversial. One group presented a positive correlation with T category and TNM stage [21], whereas other investigators showed AdipoR2 to be inversely related to T category, N category, tumor stage and lymphovascular invasion [22, 23]. Interestingly, GLO-I was directly related to AdipoR2, while no association with AdipoR1 was revealed.

We additionally examined the differential expression of AdipoR1, AdipoR2, AGE, RAGE and GLO-I between mucinous and non-mucinous tumours. We found a trend towards higher expression of all these markers except for Rage, in the subgroup of mucinous carcinomas which, although of borderline significance, seemed to be more prominent for AdipoR1 and AGE. To our knowledge, this is the first investigation of mucinous CRC, as far as the above markers are concerned.

Finally, in the present cohort, survival analysis failed to reveal AGE or RAGE as prognostic factors, in contrast to an earlier report where Dukes’ B and C cases showed a significantly poorer prognosis for RAGE positive patients [33]. Intriguingly, GLO-I expression was proven to be an aggravating factor, even after adjustment for TNM and AdipoR2, and despite the association with low T category. These findings were validated in an independent set of patients. After stage stratification, the prognostic effect of GLO-I was maintained for early stages, for both patient and validation cohorts, supporting a substantial role of GLO-I in CRC progression, most probably associated with cell detoxification from glycation by-products, such as methylglyoxal. It seems that CRC overexpressing GLO-I gain an advantage by escaping methylglyoxal-induced apoptosis resulting in tumor aggressiveness and low patients survival rate. Considering the recent glyoxalase inhibitors development [14], our findings bring forward CRC as a potential candidate for targeted therapy.

In addition, patients with high levels of AdipoR2 expression had a significantly shorter survival, which remained after stage stratification for early stage patients, as well as in multivariate analysis. This finding was somewhat expected since AdipoR2 immunoexpression was more prominent in grade III tumors. This is the first study in which expression of Adiponectin receptors is related to survival, as previous studies have failed to demonstrate such a significant association [22]. Interestingly, Baressi et al. [20] examined gastric cancer cases and noted AdipoR1 positivity to be related to significantly longer survival, although multivariate analysis did not reveal AdipoR1 as an independent prognostic factor. More extended surveys are needed to clarify the association of adiponectin receptors with patient’s survival. More importantly, our results point towards distinct roles of AdipoR1 and R2 in CRC, relating the first one with a favorable and the second with an aggressive phenotype.

## Conclusions

In the present study, we provide novel evidence regarding the possible interactions between the components of the AGE-RAGE axis, GLO-I and adiponectin receptors in CRC. Interestingly, the examined molecules seem to exert diverse roles in colorectal carcinogenesis, whereas AdipoR2 and particularly GLO-I expression are introduced as potential novel independent prognostic biomarkers of adverse significance for patients with early stage disease. Further studies are warranted to extend our observations, as well as to investigate their potential therapeutic significance.

## Abbreviations

AdipoR: Adiponectin receptor
AGEs: Advanced glycation end products
CML: Carboxymethyl lysine
CRC: Colorectal carcinoma
GLO: Glyoxalase
HMGB1: High-mobility group box-1
PRR: Pattern recognition receptor
RAGE: Receptor for advanced glycation end products
